# Respiratory surveillance through point of care molecular testing and its impacts on nursing home RSV, influenza, and SARS-CoV-2 awareness and reporting behavior: a pilot study.

**DOI:** 10.1017/ash.2026.10409

**Published:** 2026-05-28

**Authors:** H. Edward Davidson, Lisa F. Han, Kevin W. McConeghy, Ivis Perez, Elizabeth A. Sillman, Clare Nugent, Tiffany Wallace, Laurel Holland, Evan Dickerson, David H. Canaday, Stefan Gravenstein, Yasin Abul

**Affiliations:** 1 Insight Therapeutics LLC, Norfolk, VA, USA; 2 Veterans Affairs Providence Health Care System, Providence, RI, USA; 3 Health Services, Policy & Practice, Brown University School of Public Health, Providence, RI, USA; 4 Brown University Health, Providence, USA; 5 Case Western Reserve University, USA; 6 Louis Stokes Cleveland VA Medical Center, USA; 7 Warren Alpert Medical School of Brown University, Providence, RI, USA

## Abstract

**Objectives:**

To evaluate feasibility of establishing syndromic respiratory virus surveillance in a network of U.S. nursing homes, using CLIA-waived point-of-care (POC) molecular PCR testing, with centrally delivered results.

**Design:**

Single-arm feasibility pilot study.

**Setting and Participants:**

Twenty-three nursing homes (NHs) in six U.S. states, representing approximately 2,500 residents.

**Methods:**

A CLIA-waived POC molecular PCR testing device was implemented for on-site testing of respiratory syncytial virus (RSV), severe acute respiratory syndrome coronavirus 2 (SARS-CoV-2), and influenza A and B from collected nasal swabs. Symptom data was entered at the time of testing. Test results were transmitted to a centralized, web-based dashboard providing near real-time surveillance from November 2023 to January 2025. Surveys of NH staff assessed device usability, workflow integration, impact on testing procedures, and infection and outbreak awareness.

**Results:**

The established syndromic respiratory virus surveillance network conducted 1,745 point-of-care tests between November 2023 and January 2025, identifying 360 infections, including 160 COVID-19, 115 influenza A and B, and 81 RSV infections. Symptom data was captured for 99.5% of tests. Implementation was associated with high data completeness and integration into facility testing workflows. Staff reported an impact on testing protocols, infection awareness, and time to diagnosis.

**Conclusions:**

Implementation of a CLIA-waived POC molecular testing device with centralized reporting was feasible across a geographically diverse NH network. The system achieved high data capture, identified substantial viral activity, and demonstrated value for clinical management using real-time diagnostic results. This approach can be adapted for local, regional, or national real-time reporting or future pragmatic interventional studies.

## Introduction

The COVID-19 pandemic challenged nursing home (NH) care providers to develop strengthened procedures for infection control in nursing homes (NHs). Despite these advances, proactive management of respiratory virus outbreaks in NHs remains challenging due to limitations in early detection and routine surveillance. In the U.S., NH respiratory infection surveillance relies primarily on reporting to the Centers for Disease Control and Prevention (CDC) through the National Healthcare Safety Network (NHSN). Medicare-certified NHs are required to submit regular reports on case counts, vaccination status, and hospitalization data related to COVID-19, influenza, and now, respiratory syncytial virus (RSV). While these systems provide valuable population-level monitoring, the data provided by these systems do not reflect real-time viral activity at the facility level and are therefore insufficient to guide timely clinical or infection control decisions, especially in the case of RSV where testing is uncommon.

These surveillance limitations affect a highly vulnerable population of approximately 1.3 million NH residents living in over 15,000 Medicare-certified facilities nationwide.^1^ Real-time, real-world data can address this deficit and inform development of preventive measures that improve patient outcomes.

In this study, we evaluated the feasibility of implementing syndromic respiratory virus surveillance in a network of US NHs, using a CLIA-waived point-of-care (POC) molecular testing platform with symptom documentation and cloud-based, central reporting to document temporal and facility-level distribution of influenza, SARS-CoV-2, and RSV infections. High-sensitivity molecular POC testing could reduce a barrier to testing and increase test utilization and disease detection. We also hypothesized that increased testing with high-sensitivity detection would increase awareness of virus prevalence and coincide with increased vaccine uptake. Monthly surveys captured staff impressions regarding challenges, facility protocol adaptations, and the perceived value of POC molecular testing technology.

## Methods

We piloted the feasibility of implementing a CLIA-waived molecular POC device for routine respiratory virus testing in a convenience sample of U.S. Medicare-certified NHs that had at least 50 long-stay beds. The locations were chosen based on proximity to the investigators and collaborators, had leadership supportive of the study, and contracted to participate. Recruitment and analysis for this study were conducted at the facility level. All tests performed were considered eligible if they occurred in one of the participating facilities. Each facility deployed a CLIA-waived, molecular POC device (Cepheid® GeneXpert Xpress) for syndromic surveillance and detection of influenza A, influenza B, SARS-CoV-2, and RSV. Each facility received sufficient test kits and nasal swabs for testing and contact tracing, as well as test controls. Facility staff received manufacturer-led training about the device operations, routine maintenance, and use of quality control materials. Training included proper technique for loading test cartridges with biological samples and entering symptoms using the study-specific interface. These training sessions, which took approximately 30 minutes, were followed by a skill check using control cartridges. Training continued until competency of trainees was reached. These training sessions were a critical component of our data quality efforts, since data collection in pragmatic studies can present many challenges for data quality.

Nursing home (NH) staff performed testing with the CLIA-waived molecular POC devices as part of routine standard of care, following facility infection control protocols. Common provider-directed testing triggers included symptoms of influenza-like illness or contact tracing. As part of infection control measures, some facilities also used the POC device to test staff who agreed to be tested.

For each test, trained clinical staff collected an anterior nasal swab, loaded the sample to a test cartridge according to manufacturer instructions, and entered predefined symptom codes into the device interface. After approximately 34 minutes, the device displayed on-screen results that were available for download with symptom codes from a centralized, cloud-based database. The anonymized data was available to the study team and each facility’s point of contact (usually the infection preventionist). The cloud-based application was maintained by the manufacturer, segregated by facility, and contained only deidentified individual test results.

Facilities maintained a paper log of all tests performed. Printed test reports were available directly from the device, and aggregated summaries were provided back to the facilities upon request.

Participating NHs provided monthly aggregated data through a separate, secure web-based portal developed for the study. Reported data included resident and staff census, vaccination rates for influenza, SARS-CoV-2, and RSV, and facility-level respiratory virus activity. Vaccination data were derived from routine facility documentation used for regulatory reporting. Facilities also recorded the number of new cases of influenza-like illness and laboratory-confirmed influenza, COVID-19, or RSV identified each month in the web-based study portal. Monthly reports also included staff feedback on the workflow integration of the molecular testing device.

Feasibility outcomes included sustained device utilization, completeness of symptom data entry, integration into clinical workflows, staff-reported usability, and alignment between molecular test results and facility-reported cases.

Descriptive statistics were used to summarize facility characteristics, testing activity, pathogen detection, symptom documentation completeness, and survey responses. Facility-reported respiratory virus cases were compared with molecular POC–detected infections to evaluate alignment and potential reporting gaps over time. Differences in RSV case reporting between early and late phases of POC device integration were assessed using chi-square tests. Survey responses were summarized at the facility level and evaluated longitudinally to assess usability, workflow integration, and perceived clinical impact. All analyses were conducted using aggregated, de-identified facility-level data.

Compensation was provided at the facility level for study activities beyond the scope of regular clinical care with a one-time administrative fee for tasks associated with contracting, a fee for facilitating and logging manufacturer-led device-use training, and a fee for each monthly report entered into the web-based study portal.

The WCG Institutional Review Board approved this study (WCG:210234056).

## Results

### Utilization and infections detected

**Figure 1.**
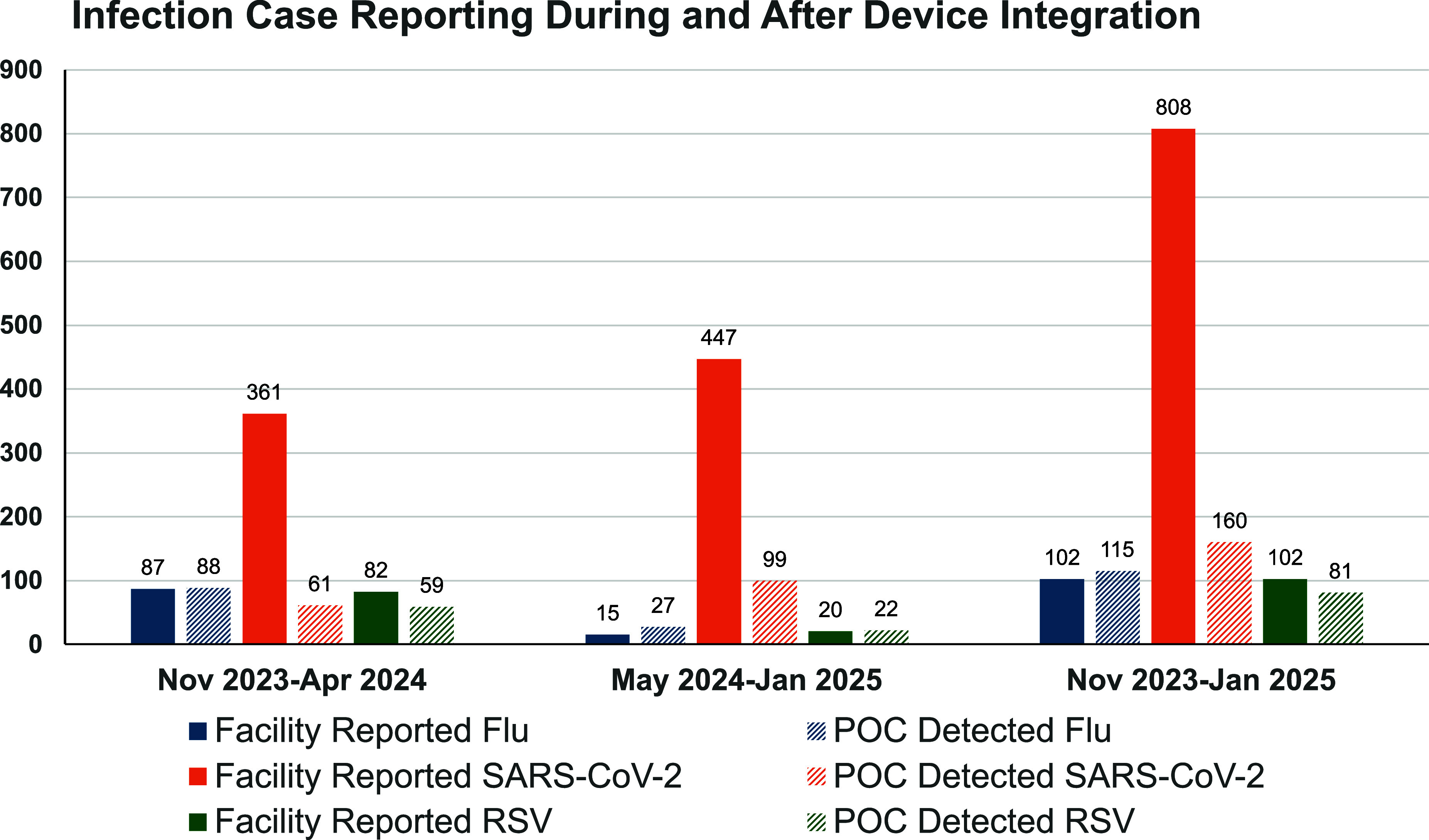
Case reports during POC testing device integration (November 2023 to April 2024) and after (May 2024 to January 2025).

A syndromic respiratory virus surveillance network was established across 23 NHs in six states (Ohio, Missouri, Indiana, Rhode Island, Virginia, and Pennsylvania). Participating facilities had an average bed count of 156 residents (range 50–427) and an average census of 130 residents (range 48–324). Across the network, the surveillance cohort represented approximately 2,520 residents. Between November 2023 and January 2025, NH staff performed 1,745 point-of-care molecular tests as part of routine clinical care, demonstrating sustained device utilization throughout the 15-month surveillance period. Network-wide, the POC molecular device identified single-pathogen infection in 360 samples. Of these, 160 cases were SARS-CoV-2s, 115 cases were influenza A or B, and 81 cases were RSV (Table 1). Co-infection with multiple pathogens was detected in 4 samples. Using the web-based study portal, facilities reported 1,012 respiratory virus cases, identified through routine clinical care using the PCR device, external laboratory PCR, and rapid antigen tests (Figure 1). SARS-CoV-2 accounted for the majority (n = 808), followed by influenza (n = 102) and RSV (n = 102).

**Table 1.** Infection case reporting during and after device integration

	Nov 2023–Apr 2024		May 2024–Jan 2025	
	Reported by facilities	Detected by molecular POC device	Reported by facilities	Detected by molecular POC device
Flu	87	88	15	27
COVID	361	61	447	99
RSV	82	59	20	22

### Data completeness

Symptom data was captured for 99.5% of tests. Some follow-up was required to reach this level of completeness, but feasibility was demonstrated regarding symptom monitoring, entry into devices, and cloud-based symptom reporting.

### Workflow integration

In monthly surveys, staff provided feedback on implementation of the POC molecular testing device in their facility. In the first six months (December 2023 to May 2024), reports that the machine was easy to use and maintain and that the user guide and testing logs were user-friendly rose from 82% of facilities to 100% (Table 2). Open-ended responses indicated that, in general, NH staff liked having access to a highly sensitive POC molecular testing device and appreciated its ability to test four viruses on-site. Staff observed that on-site testing alleviated the need to wait for lab results from an outside facility, and multi-antigen testing reduced the need to collect multiple nasal samples for rapid single-antigen testing.

**Table 2.** Staff views on point-of-care testing implementation

	Dec 2023 (n = 22) (%)	Mar 2024 (n = 21) (%)	Jun 2024 (n = 21) (%)	Sept 2024 (n = 19) (%)	Dec 2024 (n = 19) (%)	Jan 2025 (n = 19) (%)
**Overall satisfaction**						
Likely to recommend device (Greater than 5 on a 10-point scale)	68.2	85.7	100.0	89.5	89.5	89.5
**Device and protocol**						
Device is easy to use	81.8	100.0	100.0	100.0	–	–
Quick reference guide is easy to understand	81.8	90.5	100.0	100.0	–	–
Easy to enter symptoms into device	77.3	85.7	100.0	94.7	–	–
Staff is trained effectively to use the machine	72.7	85.7	90.5	89.5	–	–
Easy to maintain written testing log	77.0	86.0	100.0	89.0	–	–
**Perception and awareness**						
Changed perception of respiratory diseases	–	33.0	–	68.4	–	–
**Operational integration**						
Changed testing protocol for viruses	–	–	81.0	78.9	63.2	79.0
**Clinical impact**						
Device improved patient care^a^	–	–	–	–	89.5	89.5
Reduced time to diagnosis^b^	–	–	–	–	73.7	63.2
Improved workflow^c^	–	–	–	–	73.7	73.7

*Note:* Not all questions were asked in every survey period. Missing values (–) indicate the question was not included in that month’s survey. All percentages represent affirmative responses to the survey question.

a Combined “Very significantly improved” and “Somewhat improved” responses.

b Combined “Significant reduction” and “Slight reduction” responses.

c Combined “Improved significantly” and “Improved somewhat” responses.

### Clinical impact

By June 2024, staff from more than 80% of facilities reported having modified their testing protocols. Reported changes included using the POC molecular testing as the first line test for symptomatic residents and confirming negative rapid antigen results in high-suspicion cases. This number stayed relatively consistent, suggesting that staff from about 20% of facilities could not, or would not, adjust their established testing procedures. In January 2025, only 2 of 19 responding facilities indicated that the device did not lead to improvements in care. One indicated that the device was easy to use but noted that the reporting requirements tied to study participation negatively affected their overall experience.

We observed a statistically significant increase in RSV case reporting by staff between the early- and late-POC device integration periods, which coincided with when use of the device became routine in the later phase of the study (χ^2^ = 24.75, *P* < .001) (Figure 1). In the initial surveillance phase (Nov. 2023–Apr. 2024), NH staff reported 17 cases of RSV, while the POC molecular device detected 59 individuals with RSV. In total, by study end, staff reported more RSV cases (n = 102) than detected through molecular testing (n = 81).

**Figure 2.**
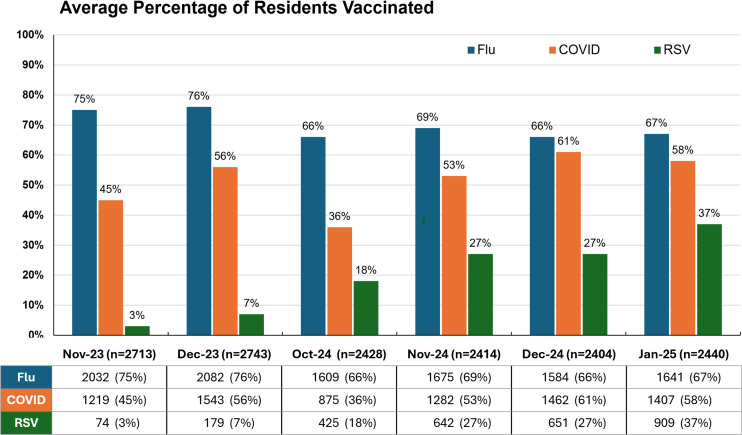
Percentage of vaccine uptake for influenza, SARS-CoV-2, and RSV at participating NHs during POC testing phases.

The percentage of SARS-CoV-2 and RSV vaccine uptake among residents in participating NHs also increased as use of the POC testing for clinical care became routine, while the already high influenza vaccine uptake remained relatively stable (Figure 2).

## Discussion

This study adds scientific knowledge by demonstrating the feasibility of sustained molecular point-of-care respiratory surveillance in NHs and by showing that improved diagnostic sensitivity alters RSV detection and reporting behavior. It also provides real-world implementation insights into how molecular testing is integrated into existing workflows and suggests that real-time virus awareness may influence preventive practices such as vaccination.

Implementation of a CLIA-waived POC molecular testing device with centralized reporting was feasible across a geographically diverse NH network. Facilities sustained device utilization over a 15-month period, achieved near-complete symptom data capture, and integrated testing into routine clinical workflows with minimal reported disruption. These findings demonstrate that molecular POC testing with centralized data aggregation can support real-time respiratory virus surveillance in long-term care settings.

In participating facilities, molecular POC testing complemented rather than replaced existing testing strategies. Staff continued to rely on rapid antigen tests during outbreak situations where high-volume screening was operationally necessary, while molecular testing was preferentially used to confirm cases, clarify ambiguous results, and guide early clinical decision-making. This hybrid approach reflects real-world staffing and cost constraints in NHs and highlights how molecular testing can add value without displacing established practices. Importantly, molecular testing provided higher diagnostic confidence at symptom onset, when rapid antigen tests might yield false-negative results.

Cost considerations are an important factor in the implementation of molecular point-of-care testing in NHs. Molecular POC platforms such as the one used in this study require up-front capital investment as well as ongoing per-test cartridge costs that are higher than those of rapid antigen tests. In this pilot, testing devices, cartridges, and training were supported through study funding, and costs were not directly evaluated. However, facilities reported operational benefits including faster time to diagnosis, reduced need for confirmatory testing, and improved clinical decision-making, which may offset some costs in practice. Future studies should formally evaluate cost-effectiveness and reimbursement strategies to determine the sustainability of molecular POC surveillance in long-term care settings.

The statistically significant increase in RSV case reporting by staff between the early- and late-POC device integration periods suggests that use of molecular POC testing in NHs may improve early RSV detection, increase awareness and accuracy of RSV reporting, thereby enhancing existing surveillance practices by reducing underreporting through better detection and more timely data capture. Continued integration of testing devices and staff training may further close the reporting gaps and strengthen RSV outbreak recognition. Furthermore, the molecular device could prove useful in confirming or countermanding negative rapid antigen test results, reducing concerns about false negatives and their much lower sensitivity. The molecular device also reduced patient discomfort and simplified the test process by simultaneously testing for multiple respiratory pathogens (COVID-19, influenza A and B, and RSV) from a single anterior nasal sample.

Findings from the pilot study demonstrated the feasibility of implementing point-of-care molecular testing and documentation in a NH, with reliable device performance and robust operational execution.

By supporting timely clinical decision-making and targeted infection control interventions, this approach can offer a significant advancement in respiratory virus monitoring for vulnerable populations, potentially transforming surveillance strategies in long-term care settings.

Accurately confirming or excluding influenza, SARS-CoV-2, or RSV infections help prevent unnecessary isolation, which negatively affects both residents and staff in long-term care settings.^2–6^ Reducing unnecessary isolation of uninfected individuals therefore remains a priority.

Real-time molecular POC testing may support more precise infection control decisions by enabling rapid exclusion of infection while also generating high-quality, facility-level surveillance data that can be aggregated across facilities. Monthly facility surveys tracked the implementation and perception of point-of-care molecular testing technology across the NH network and demonstrated a progressive improvement in staff acceptance and operational integration of the device. Facilities that withdrew from the study did so primarily because of administrative changes, acquisitions, or staffing shortages, rather than because of study demands or a perceived lack of device value. According to facility leadership, the most common operational challenge was the need for ongoing training and retraining of frontline staff, reflecting both high turnover rates, typical in the long-term care environment, and the integration of new testing processes. One solution observed across facilities was the designation of one or two trained individuals, most commonly the Infection Preventionist or a designated nurse to perform testing, rather than attempting universal training of all clinical staff.

Prior research shows that many NHs lack the funding and infrastructure to support sustained health information technology (HIT) implementation,^7^ with approximately 28% experiencing system abandonment within a 2-year period.^8^ Smaller facilities, in particular, often have lower HIT capacity,^9^ but despite this variability, the high level of acceptance observed in this study suggests that molecular POC testing can be successfully adopted in NHs when appropriate training, operational support, and perceived clinical relevance are present.

Staff widely recognized the technology’s benefits, which was reflected in their high recommendation rate for the adoption of the technology. A literature review conducted in 2018 found that front-line staff often reported that HIT reduces time with residents and has minimal influence on clinical decision making, but since implementation of widespread technology use during the COVID pandemic, staff may more readily accept new technology as a means to improve care.^7^

We also observed a potential side benefit of having real-time respiratory virus detection: improved uptake for both SARS-CoV-2 and RSV vaccines. Had only RSV vaccine uptake increased, we would have ascribed the change to the expected increase in vaccine use after its initial introduction. It appears that on-site, real-time virus detection raises staff and resident awareness of infection risk and supports informed communication on prevention, including vaccination.

By utilizing a CLIA-waived POC molecular PCR testing platform that sends data via the cloud, we paired POC testing with the ability to visualize testing results in real-time at a central location. Our data indicates that implementing molecular POC testing led to changes in how NH staff reported respiratory viruses and coincided with increased vaccine uptake in residents. Further, we also captured critical insights into frontline practitioners’ perspectives on this emerging technology and their ability and willingness to adopt it as a new standard.

The study validated the practical utility of POC molecular testing in NH environments. The POC device’s real-time diagnostic capabilities enable rapid identification and response to respiratory virus outbreaks. Moreover, the granular data collected offers a scalable approach for a potentially transformative resource that could support national seasonal virus tracking and predictive modeling, bridging the gap between localized infection control and broader public health surveillance.

This model offers an approach for improving infectious disease monitoring in other congregate living settings, such as assisted living facilities, rehabilitation centers, and group homes. Virus surveillance networks utilizing POC molecular testing in NH settings may help mitigate the impact of future pandemics by reducing underreporting and enhancing awareness of respiratory pathogens among staff and residents. This increased staff awareness may also ultimately positively influence vaccine acceptance among residents and staff.

Our research also establishes a framework for epidemiological investigations in long-term care facilities. By highlighting discrepancies between facility-reported and molecular-confirmed infections, the study reveals key challenges in virus detection. The developed infrastructure offers a reproducible model for surveillance in vulnerable populations and enables more nuanced temporal and geospatial analyses. Future research could extend this model to other confined environments, transforming our understanding of pathogen transmission and the impact that POC molecular testing may have on staff and resident vaccine uptake.

The study underscores the potential of a syndromic surveillance network in NHs to turn reactive outbreak management into proactive prevention and serve as an early detection model for pandemics. Technology-enabled surveillance could reduce hospitalizations and protect older adults. Federal and state agencies should prioritize POC molecular testing in NHs, including IT infrastructure for data extraction, secure transmission, and staff training.

## Manuscript preparation

Moderna collaborated with the study team in the design and methods and offered suggestions to the final manuscript.

## Brief summary

A pilot study in 23 NHs showed POC molecular testing (1,745 swabs; 360 infections) improved reporting, awareness, and speed of diagnosis, proving feasibility for broader surveillance.

## Previous presentation

Portions of this work have previously appeared in abstract form:

- Davidson HE, Han L, McConeghy K, et al. A pilot study to establish a nursing home syndromic surveillance network. Open Forum Infectious Diseases. 2025;12(Suppl 1):ofae631.2483. doi:10.1093/ofid/ofae631.2483.
- Davidson HE, Han L, McConeghy K, Abul Y, et al. A pilot study to establish a nursing home syndromic surveillance network. Open Forum Infect Dis. 2025;12(Suppl 1):S1367.
- Abul Y, Davidson HE, Han L, et al. Bridging the gap in RSV detection: nursing home respiratory pathogen detection and reporting with enhanced surveillance. Am J Respir Crit Care Med. 2025;211:A4203. doi:10.1164/ajrccm.2025.211.Abstracts.A4203.

These abstracts describe preliminary findings that are expanded upon in the present manuscript. No full-length manuscript containing these data has been published or is under consideration elsewhere.
